# Treatment of Gastrointestinal Stromal Tumors (GISTs): A Focus on Younger Patients

**DOI:** 10.3390/cancers14122831

**Published:** 2022-06-08

**Authors:** Monika Dudzisz-Śledź, Anna Klimczak, Elżbieta Bylina, Piotr Rutkowski

**Affiliations:** Department of Soft Tissue/Bone Sarcoma and Melanoma, Maria Sklodowska-Curie National Research Institute of Oncology, Roentgena 5, 02-781 Warsaw, Poland; anna.klimczak@pib-nio.pl (A.K.); elzbieta.bylina@pib-nio.pl (E.B.); piotr.rutkowski@pib-nio.pl (P.R.)

**Keywords:** gastrointestinal tumors, GIST, young adult, TKI, tyrosine kinase inhibitor, wild-type, KIT, PDGFRA, NF1, SDHB, SDH-competent, SDH-deficient

## Abstract

**Simple Summary:**

Gastrointestinal stromal tumors (GISTs) are the most common mesenchymal neoplasms of the gastrointestinal tract. GISTs mainly develop in older adults, and the median age of diagnosis is 60–65 years. The incidence of GISTs in young adult patients, defined as adults before 40, is less than 10%. The frequency and type of molecular abnormalities in this group of patients are different from those in older patients. In this publication, we focus on the specificity of GISTs in young people and the principles of therapeutic management and management of the side effects of treatment.

**Abstract:**

Gastrointestinal stromal tumors (GISTs) originate from Cajal’s cells and are the most common mesenchymal neoplasms of the gastrointestinal tract. GISTs in young adults, i.e., patients before the age of 40, are rare and differ from those in older patients and GISTs in children in terms of the molecular and clinical features, including the location and type of mutations. They often harbor other molecular abnormalities than *KIT* and *PDGFRA* mutations (wild-type GISTs). The general principles of therapeutic management in young patients are the same as in the elderly. Considering some differences in molecular abnormalities, molecular testing should be the standard procedure to allow appropriate systemic therapy if needed. The optimal treatment strategy should be established by a multidisciplinary team experienced in sarcoma treatment. The impact of treatment on the quality of life and daily activities, including the impact on work, pregnancy, and fertility, in this patient population should be especially taken into consideration.

## 1. Introduction

Gastrointestinal stromal tumors (GISTs) usually develop in older people, and the median age of diagnosis is 60–65 years. GISTs rarely develop in younger patients. In children, GISTs often occur in girls, are located in the stomach, and generally do not have *KIT/PDGFR* mutations. The typical phenotypic and genotypic patterns in young adults aged 18 to 40 years are unknown. Less than 10% of GISTs are diagnosed in young people, i.e., before 40, and less than 1% of GISTs are diagnosed below 21 [1]. This disease in this population must be appropriately managed to optimize the efficacy and tolerability, primarily due to the long-expected survival and active participation in social and family life and the disease’s impact on work, psychological aspects, and fertility. Young adulthood is a period of significant physical and psychosocial change, including continuing education, gaining financial independence, entering romantic relationships, starting a family, and raising children [2]. The disease and its treatment can interfere with daily activities and make it difficult to carry out daily activities. The treatment strategy should be defined by a multidisciplinary team experienced in soft tissue sarcomas, comprising an oncological surgeon, medical oncologist, pathologist, radiologist, gastroenterologist, and nuclear medicine specialists. Surgical treatment with R0 resection (negative margins), if possible, remains the mainstay of GIST management.

In some cases, preoperative therapy may be introduced. High- and intermediate-risk GISTs require adjuvant therapy [3]. For metastatic disease, targeted therapies are available, but surgery may also be used in some cases. Due to the molecular characteristics of young adult GISTs, specific considerations regarding appropriate therapy are needed to introduce the optimal treatment in young individuals with GISTs.

## 2. Epidemiology

GISTs are the most common mesenchymal neoplasms of the gastrointestinal tract. The incidence has been increasing during the last decades and, in most published studies, is reported at 10–15 new cases/per 100,000 per year [4,5,6,7].

## 3. Biology and Molecular Biology

**Figure 1:**
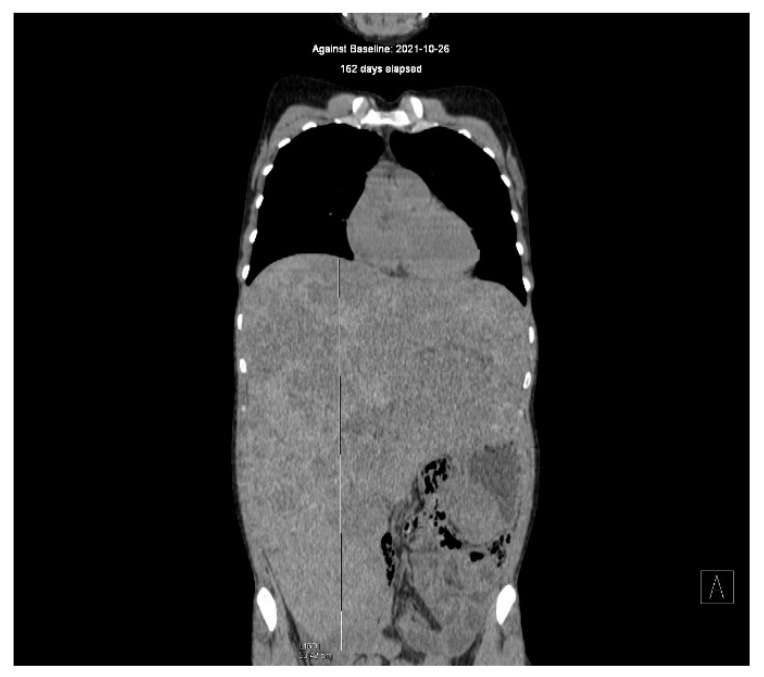
Advanced wild-type GIST originating in the stomach in young adult women treated for 18 years.

The most common location of GISTs is the stomach (~60%). Less often, they are located in other parts of the gastrointestinal tract: about 30% of GISTs are detected in the jejunum and ileum, 5% in the duodenum, 5% in the rectum, and <1% in the esophagus [1]. Most GISTs are detected due to symptoms, but some are diagnosed as incidental findings during surgery or autopsy. The median size of GISTs ranges from 2.7 to 8.9 cm [5]. An example of advanced wild-type GIST originating in the stomach in young adult women treated for 18 years is shown in Figure 1.

Two typical histological patterns of GIST are known. One is the spindle cell (60–70%) pattern and the second is epithelioid (30–40%). In some cases, a combination of both patterns in variable proportions occurs. GISTs stain positive for KIT (CD117, 95%) and DOG1 (almost exclusively characteristic for GIST). DOG1 expression is independent of the KIT status. About 5% of GISTs are CD117 negative, mainly in patients with the *PDGFRA* mutation [8].

Abraham et al. studied 150 esophagogastric resections for esophageal or esophagogastric junction carcinomas, and they found incidental GISTs in 15 of 150 (10%) patients [9]. All detected GISTs had a spindle cell morphology and were positive for CD117 and CD34. A study conducted in Germany showed a 25% incidence of small GISTs in the stomach in autopsies [10]. The majority of GISTs are sporadic and solitary, and about 80% of GISTs harbor activating mutations in the *KIT* or *PDGFRA* genes. These genes are responsible for the upregulation of crucial signaling pathways, including MAPK and PI3K-AKT. Up to 20% of CD117 (+) GISTs do not harbor *KIT* and *PDGFRA* mutations, i.e., are wild-type GISTs [11]. *PDGFRA*-mutant tumors are primarily located in the stomach, mesentery, and omentum. GISTs with *KIT* exon 9 mutations primarily develop in the small intestine [12]. Most GISTs in children are wild type. 

GISTs in young adults differ from pediatric GISTs and are not similar to typical GISTs in older patients. The data about the biology of GISTs in young adults is limited. IJzerman et al. published data from the Dutch GIST Registry for young adults aged 18 to 40 years and compared this data with data from older patients (>40). Of 1010 patients, 52 patients were ≤40 years. The authors found statistically significant differences between young and older GIST patients regarding the localization, mutational status, and presentation. The tumors were primarily located in the stomach (46%) and small intestine (46%). *KIT* mutations were diagnosed in 69% of patients and *PDGFRA* in 6%. In total, 25% of patients did not harbor *KIT* and *PDGFRA* mutations. Among them, 8% had *SDH*-deficient disease, 4% associated with NF1, 2% with *ETV6-NTRK3* gene fusion, and 10% were wild type. Young patients with GISTs were more often diagnosed in an emergency setting (18% vs. 9%). The overall 5-year survival rate was 85% [13].

Kang et al. analyzed 22 cases of GISTs in children and young adults up to 30 years of age. Of the 20 GISTs in young adults, 60% were located outside the stomach, and *KIT* or *PDGFRA* gene mutations were identified in 78% of the 18 cases. Ninety patients underwent R0 resection. One patient with a GIST located in the small intestine and with the *KIT* exon 11 deletion mutation had recurrent disease and was treated with imatinib with partial response. At diagnosis, one patient with multiple GISTs located in the stomach and with perigastric lymph node metastases developed multiple distant metastases and died after 7.3 years [14].

GISTs in young adults may have GIST features of child or adult GISTs. Prakars et al. analyzed the clinicopathologic and molecular features of 15 cases of GISTs in children and young adults (<30 years old). They included 15 patients with GIST, 5 children and 10 adults. Half of the 10 GISTs in young adults occurred in the small intestine and had a spindle cell morphology. In one case, lymph node metastasis was found. *KIT* mutations were identified in seven cases, four in exon 11 and three in exon 9. Recurrence was observed in seven patients [15].

Advances in molecular biology have allowed recognition that GISTs without *KIT/PDGFRA* mutations are usually deficient in succinyl dehydrogenase (SDH) due to silencing the epigenetic *SDHC* gene, and/or they have mutations in *NF1* and *BRAF* V600E, or *NTRK* gene rearrangement [16,17].

*SDH* mutations are more frequent in younger patients, especially in GISTs arising from the stomach. SDH-deficient GISTs make up 5% to 7.5% of all GISTs. The GIST with *SDH* mutations tends to metastasize, may metastasize to lymph nodes (which is unusual in typical GISTs), less frequently metastasize to the liver, usually grow slowly, and are often resistant to imatinib. In histological examination, these tumors are characterized by a multinodular growth pattern with epithelioid cells, which are multifocal. Additionally, it was found that SDH-deficient GISTs overexpress insulin-like growth factor receptors (IGF1R) [18]. Testing for germline mutations in *SDH* should be considered in young adults with GISTs wild type for *KIT/PDGFRA* mutations.

Some clinical syndromes are associated with GISTs, and these cases are usually diagnosed in young adults. One of them is the Carney’s triad, which is usually a sporadic association of pulmonary chondroma, GISTs, and paraganglioma. GISTs in Carney’s triad differ clinically, pathologically, and behaviorally from sporadic GISTs. Zhang et al. studied the clinical and pathologic features of the gastric neoplasm in 104 patients with Carney’s triad. They found that GISTs in Carney’s triad mainly affect young (mean age 22) women (88%), are often multifocal with higher epithelioid cell predominance, more often metastasize to lymph nodes, and often relapse. Their behavior is unpredictable [19]. The usual presentation was gastric bleeding. The second one, Carney-Stratakis syndrome, is a combination of familial paraganglioma and GISTs and is usually inherited and not associated with pulmonary chondroma. It is difficult to distinguish between Carney’s triad and Carney-Stratakis syndrome due to the rarity of the components, and molecular testing may be helpful. GISTs in Carney-Stratakis syndrome and Carney’s triad may be SDH-deficient GISTs. Carney’s triad is usually caused by a specific pattern of methylation of the *SDHC* gene and may be due to germline mosaicism of the responsible genetic defect. Carney-Stratakis syndrome is instead caused by inactivating germline mutations in genes encoding for the SDH subunits [18].

In addition, type 1 neurofibromatosis (NF1), resulting from a loss-of-function mutation in the *NF1* gene, may be related to multifocal GIST, predominantly located in the jejunum or ileum, with the frequent presentation of gastrointestinal bleeding and anemia [20].

Based on the information above, primary resistance to imatinib is more common in GISTs in young populations due to the presence of mutations that prevent the molecule from binding to its KIT- and PDGFRA-binding sites. Wild-type GISTs are commonly insensitive to standard therapies, including imatinib [21,22,23]. About 10–15% of patients develop primary and early resistance during the first six months of therapy. The other most common genetic abnormality associated with primary resistance to imatinib is the D842V mutation, which relies on substitution of aspartic acid in codon 842 of PDGFRA into valine. Moreover, GISTs with mutations in exon 9 show a lower response rate and progression-free survival to imatinib than the most common exon 11 mutations when imatinib is used at a dose of 400 mg/day. In such cases, imatinib used at a dose of 800 mg/day is associated with better progression-free survival.

In addition to primary resistance, secondary resistance to imatinib may occur. The disease progresses in approximately 40–50% of patients during the first 2–3 years of imatinib therapy. This secondary resistance may be due to the accumulation of secondary point mutations in different regions of the *KIT* and *PDGFRA* genes [24]. It may also be due to the fibroblast growth factor (FGF) and the FGF receptor (FGFR) [25]. It has been shown that crosstalk between KIT and FGFR can promote imatinib resistance by reactivating the MAPK signaling pathway. Imatinib resistance may be promoted by crosstalk between KIT and FGFR due to the reactivation of the MAPK signaling pathway.

Hostein et al. analyzed a series of 321 GISTs for *BRAF* mutations and B-raf expression. They analyzed 251 GISTs with *KIT* or *PDGFRA* mutations and 70 wild-type GISTs. Among GISTs with *KIT* and *PDGFRA* mutations, no V600E mutation was detected. In wild-type GISTs, nine cases were positive for V600E mutation. GISTs with *BRAF* mutations were mainly localized in the small intestine and the stomach. No statistical difference in tumor location and other histologic and clinical features, including age, was found between WT GISTs with or without *BRAF* mutations. Three patients with *BRAF* mutations were high risk, three intermediate, two low, and in one case, the risk was not determined. They assessed BRAF expression in 37 GISTs (8 wild-type *BRAF* V600E-positive, 9 wild-type *BRAF*-negative, and 20 *KIT*- or *PDGRFA*-positive. BRAF expression was present in all cases. About 13% of *KIT* and *PDGFRA* wild-type GISTs are *BRAF* mutated [26].

Brenca et al. identified one fusion gene, *ETV6-NTRK3*, in one case of GIST among five *KIT/PDGFRA/BRAF* mutation-negative SDH-proficient tumors [27]. Shi et al. performed genetic comprehensive genomic profiling for coding regions in about 300 cancer-related genes of 186 GISTs to identify their somatic alterations. They found 24 GISTs without *KIT*, *PDGFRA*, and *RAS* mutations. Twelve did not harbor *SDH* alternations. The median age of patients with wild-type tumors was 44.4 years. The authors identified the most common mutated genes: *ARID1B*, *ATR*, *FGFR1*, *LTK*, *PARK2*, *SUFU*, and *ZNF217*. In two GISTs, *FGFR1* gene fusions were detected (*FGFR1–HOOK3*, *FGFR1–TACC1*), and one *ETV6–NTRK3* fusion that responded to TRK inhibition [28].

The summary of the main characteristics of GISTs in young people is presented in Table 1.

## 4. Treatment

### 4.1. Surgery and Perioperative Therapy

The young adult population is a particular group of patients. Their lifestyle, lack of routinely used screening tests, delays in presentation, lower incidence of neoplasms, and, therefore, less frequent association of symptoms with suspected neoplasms may delay diagnosis and treatment.

The data on the treatment of GISTs in young patients is limited. As with all patients with GISTs, the main goal of GIST treatment is the surgical removal of tumors with histologically negative margins (R0). Molecular testing is recommended.

It was shown, based on the National Cancer Institute’s Surveillance, Epidemiology, and End Results (SEER) database analysis of patients with GISTs, that surgical treatment in people below 40 is performed more often (84.7% vs. 78.4%, *p* = 0.003), and is related to better outcomes in terms of GIST-specific survival (GSS) and overall survival (OS) in comparison to patients ≥40, including patients with metastases. This retrospective cohort study included 392 young patients (13–39) and 5373 older adult patients diagnosed from January 2001 until December 2013, with follow-up until December 2015. Young patients were more often diagnosed with small intestine GISTs than older patients (35.5% vs. 27.3%, *p* = 0.008). In the young patient subgroup with GISTs located in the stomach and small intestine, the small intestine location was associated with improved OS (91.1% vs. 77.2%, *p* = 0.01%), and GSS (91.8% vs. 78.0%, *p* = 0.008) and were more often treated surgically (84.7% vs. 78.4%, *p* = 0.003). In general, surgical treatment improved the prognosis in young patients with GISTs. Management without surgery was associated with a more than two-fold increased risk of death from GISTs [29].

Extended anatomic resections and complex multivisceral resections should be avoided whenever possible. In low-risk GISTs located in unfavorable locations, the R1 resection may be acceptable, and the decision should be made with the patient [30]. Usually, for R1 resection, routine re-excision is not recommended on a routine basis, and the microscopic margin status should not be taken when deciding on adjuvant therapy. If extended surgery is required, imatinib neoadjuvant therapy should be considered in GISTs with imatinib-sensitive mutations.

For GISTs insensitive to imatinib, i.e., GISTs with *PDGFRA* exon 18 mutations (including the D842V mutation), neoadjuvant avapritinib may be considered. Before neoadjuvant therapy, a biopsy should be performed to confirm the diagnosis and molecular testing. Imatinib can be continued in a preoperative setting until the maximum response, with close response assessment to avoid missing the resistance and progression. Imatinib should be continued in an adjuvant setting after surgery. Patients after preoperative avapritinib should undergo observation after surgery [30]. Patients with *PDGFRA* mutations and wild-type GIST after curative surgery have a lower risk of recurrence than patients with KIT mutations [3,30].

In patients who were treated systemically perioperatively, imatinib can be stopped right before surgery and restarted as soon as the patient can tolerate oral medications. Avapritinib should be stopped at least one week prior to surgery.

During the surgery, every effort should be made not to disturb the tumor pseudocapsule. One of the adverse prognostic factors is tumor rupture.

In some circumstances, a laparoscopic (favorable anatomic locations, small tumors) or endoscopic (small tumors in the upper or lower GI tract) approach may be considered. Based on a meta-analysis of 19 studies, including 1060 patients with GISTs, it was shown that there is no difference between laparoscopy and laparotomy regarding long-term outcomes. Laparoscopy was associated with less blood loss, lower complication rates, and shorter hospitalization [31].

The incidence of nodal metastases is low, and lymphadenectomy is usually not required. Lymphadenectomy must be considered in patients with known SDH-deficient GISTs or translocation-associated GISTs and pathologically enlarged nodes. For SDH-deficient GISTs with multifocal disease, extensive surgery associated with significant morbidities, such as total gastrectomy, is not recommended to reduce the risk of recurrence in the stomach [3]. In GIST patients with SDH deficiency or known *SDH* mutations, the risk of paraganglioma is increased, and diagnostic tests should be considered prior to surgery. Patients with *SDH* mutations are at risk of paraganglioma, and they should be tested using 24-h urine collection prior to the surgery [3].

The available data indicate that perioperative imatinib should be used for three years (including preoperative therapy). As per NCCN guidelines, adjuvant imatinib is preferred for patients with a significant risk of recurrence, i.e., intermediate or high risk. The ESMO guidelines recommend adjuvant therapy for high-risk patients, and for patients with intermediate risk, the decision should be individualized, and the decision-making process may include genotyping for KIT mutations [3,30].

Three years of therapy improved the relapse-free survival (RFS 65.6% vs. 47.9%) and overall survival (OS, 5-year OS: 92% vs. 81.7%, 10-year OS: 79% vs. 65.3%) in comparison to the one-year therapy [32,33]. The phase II PERSIST-5 study has shown that five years of adjuvant imatinib therapy was associated with little risk of recurrence in patients after resection of intermediate- or high-risk GISTs [34]. The 5-year recurrence-free survival was 90%, and the 5-year overall survival was 95%; 49% of patients did not complete therapy, with the time from treatment discontinuation to relapse ranging from 7 to 24 months [35]. The ESMO guidelines recommend perioperative therapy with imatinib for three years [30].

Patients with *PDGFRA* D842V-mutated GISTs should not be treated with adjuvant therapy due to the lack of imatinib efficacy and other systemic options in adjuvant settings [30]. Adjuvant treatment, including imatinib, is not recommended in NF1-related and SDH expression-negative, *BRAF*-mutated, or *NTRK*-rearranged GISTs [30].

GIST staging is usually based on the American Joint Committee on Cancer (AJCC) TNM classification system 8th edition from 2017 [3].

The risk assessment uses tumor features such as the primary mitotic count, tumor size, and tumor site, and standard risk classification does not include mutational status [36,37].

### 4.2. Treatment of Unresectable/Metastatic GISTs

In unresectable and metastatic settings, systemic therapy with targeted therapies is the mainstay of disease management. GISTs are generally resistant to chemotherapy and radiation therapy.

Medical therapy in young adults should be simplified to adjust to daily activities, whenever possible. The visits and consultations should be flexible to enable young adults to continue therapy without impacting school or work and daily activities. The patients should be well educated about the disease, prognosis, treatment options, and additional support, including mental health specialist and psychological counseling. It should also include counseling about fertility impairment and preservation before treatment initiation and its impact on sexual health. Smokers should be referred to a smoking cessation program. Patients should also be educated on dietary recommendations and possible changes related to cancer treatment [38].

Imatinib is the standard of care in the first line of unresectable and metastatic GISTs. It was the first drug introduced into clinical practice in GISTs. It is a KIT and PDGFRA tyrosine kinase inhibitor. Before discovering imatinib, the median OS of patients with unresectable or metastatic disease was 12–15 months. Based on prospective clinical trials, imatinib improved OS to approximately five years. The median PFS was 2–3 years. Complete responses were rarely observed in 5–7% of patients, but partial responses were observed in 40% of patients, and disease stabilization in 36% [39,40,41]. These data were confirmed in clinical practice [39,42]. Imatinib is used at a daily dose of 400 mg, and the daily dose may be increased in case of disease progression to 800 mg [43]. The best responses to imatinib occur in GISTs with mutations in *KIT* in exon 11. GISTs with exon 9 *KIT* mutations and GISTs without *KIT* mutations, and GISTs with specific mutations in the PDGFRA gene, especially D842V, are less or insensitive to imatinib.

For adult patients with unresectable or metastatic GIST harboring the *PDGFRA* D842V mutation, which is resistant to imatinib, avapritinib has been approved for first-line therapy based on phase I NAVIGATOR study results. Avapritinib is a type 1 kinase inhibitor that has demonstrated activity on the *PDGFRA* D842V and *KIT* D816V mutants associated with resistance to imatinib, sunitinib, and regorafenib. The USA approved the drug to treat unresectable or metastatic GIST patients harboring *PDGFRA* exon 18 mutations, including the *PDGFRA* D842V mutation. In Europe, this drug has been approved for *PDGFRA* D842V GISTs. In patients with GISTs with the *PDGFRA* D842V mutation treated with different doses, the objective response rate (ORR) was 88%, with CR in 9% of patients, PR in 79%, and SD in 13%. The ORR among 38 patients with the *PDGFRA* D842V mutation treated with avapritinib 300 or 400 mg was 95%, with CR in 13% of patients and PR in 82%. The median PFS was 24 months. Median OS was not reached, and the duration of response was 22 months [44,45].

In the phase III VOYAGER study, avapritinib was compared to regorafenib in patients with unresectable or metastatic GIST previously treated with imatinib and one or two other tyrosine kinase inhibitors (TKIs). The primary endpoint of an improvement in PFS was not met. PFS was 4.2 months in the avapritinib arm and 5.6 months in the regorafenib arm [46].

Patients who progress or are intolerant to imatinib may be treated with sunitinib in the second-line therapy. Sunitinib is a multitargeted TKI that targets KIT receptor tyrosine kinase, PDGFR, VEGFR, and FLT3. This is the only TKI approved for GIST therapy in the second line. Up to 40% of patients on sunitinib, especially with the exon 9 *KIT* mutation, may achieve long-term responses, with the time to progression 6–8 months (median). In a phase III, randomized, placebo-controlled, double-blind study, the progression-free survival was 22.9 vs. 6.0 weeks in the placebo arm. PFS and OS were longer in patients with a primary *KIT* exon 9 mutation or wild-type *KIT/PDGRFA*. The recommended dose of sunitinib is 50 mg taken orally once daily for 4 consecutive weeks, followed by a 2-week rest period (schedule 4/2). Dose modifications in 12.5 mg steps may be applied for toxicity management based on individual safety and tolerability. An option is a continuous dosing regimen with a dose of 37.5 mg daily without interruption [47,48,49,50].

Regorafenib is another oral inhibitor that potently blocks multiple protein kinases, including kinases involved in tumor angiogenesis (VEGFR1, -2, -3, TIE2), oncogenesis (KIT, RET, RAF-1, BRAF, BRAFV600E), metastasis (VEGFR3, PDGFR, FGFR), and tumor immunity (CSF1R) [51]. The drug is approved for treatment of adult patients with GISTs who have been previously treated with other anticancer medicines (imatinib and sunitinib). The recommended dose is 160 mg taken orally once daily for the first 21 days of each 28-day cycle. Regorafenib was assessed in a multicenter phase II study [52] and phase III GRID trial [53]. In the previously pretreated population, the mean PFS in the regorafenib group was more than five times longer than in the placebo group.

Ripretinib is a TKI that inhibits KIT proto-oncogene receptor tyrosine kinase and PDGFRA kinase, including wild-type, primary, and secondary mutations, and inhibits other kinases in vitro such as PDGFRB, TIE2, VEGFR2, and BRAF. Ripretinib was assessed, in comparison to a placebo, in the phase II INVICTUS study in patients with disease progression on at least imatinib, sunitinib, and regorafenib or who had documented intolerance to any of these medications despite dose modifications. Ripretinib, as a fourth or further treatment line, significantly improved median PFS compared with the placebo (6.3 vs. 1.0 months; HR 0.15; 95% CI 0.09–0.25; *p* < 0.0001). Median OS was 15.1 and 6.6 months in the ripretinib and placebo arms, respectively (HR 0.36; 95% CI 0.21–0.62) [54].

Ripretinib has also been assessed compared to sunitinib in the phase III INTRIGUE study in patients with advanced GIST after treatment with imatinib. PFS was not statistically different between the ripretinib and sunitinib arms, and median OS was not reached in either arm [55].

A summary of the efficacy of drugs approved for treatment of unresectable and metastatic GISTs is provided in Table 2.

Pazopanib, an orally administered, potent multi-target tyrosine kinase inhibitor (TKI) of VEGFR1, VEGFR2, VEGFR3, PDGFRA, PDGFRB, and c-KIT, was assessed in the phase II PAZOGIST study in patients with GIST (*n* = 81) with failure on imatinib and sunitinib. The 4-month PFS rate in central assessment was significantly higher in the pazopanib group (44.3%) compared to the control group (17.6%). Based on the investigator’s assessment, the median PFS was 3.4 months in the pazopanib arm and 2.3 months in the placebo group [57].

Sunitinib, regorafenib, and pazopanib may be more effective in GISTs with *SDH* mutations and SDH-deficient GISTs. The multicenter series of pediatric/young adult patients with advanced *KIT/PDGFRA* WT GISTs treated with sunitinib (potent antiangiogenic inhibitor) confirmed some clinical benefits of sunitinib in this population [58]. These data were similar to the series of Janeway et al. in pediatric GIST patients. A longer time to progression on sunitinib compared to prior imatinib therapy was observed [59].

Sorafenib, a multikinase inhibitor with activity against KIT and PDGFRA and several other kinases, was assessed in two single-arm phase II clinical trials in patients with GISTs after progression during therapy with imatinib and sunitinib. Median PFS in both trials was about 5 months, and OS was 9.7 and 11.6 months, respectively [60,61].

Patients with GIST with *NTRK* rearrangement may be sensitive to the neurotrophic tyrosine receptor kinase (NTRK) inhibitors larotrectinib and entrectinib. The efficacy of NTRK inhibitors was shown in clinical trials in patients with solid tumors [30,62,63].

Patients with GISTs with *BRAF* mutations may benefit from BRAF inhibitors (including the anti-BRAF plus anti-MEK combination) [30]. Falchook et al. showed the activity of the BRAF inhibitor dabrafenib in non-melanoma solid tumors in a phase II study [64]. They also showed dabrafenib’s antitumor activity in GIST patients with the V600E *BRAF* mutation [65].

Other molecules that may be effective in some GISTs include nilotinib, ponatinib, dasatinib, cabozantinib, and crenolanib.

Nilotinib, a selective and potent TKI targeting BCR-ABL, c-KIT, PDGFR, and other kinases, is effective in patients who failed both imatinib and sunitinib due to disease progression or intolerance. In the study conducted by Montemurro et al. (*n* = 52), 10% of patients responded to nilotinib, and 37% achieved disease stabilization. Median PFS was 12 weeks, and median OS was 34 weeks [66]. In the post hoc subset analyses in the phase III study, nilotinib provided significantly longer median OS in patients pretreated with imatinib and sunitinib [67].

Ponatinib is a potent pan BCR-ABL inhibitor, which demonstrated activity against RET, FLT3, and KIT and members of the FGFR, PDGFR, and VEGFR families of kinases [68]. This drug has shown activity against the KIT exon 17 D816-mutant kinases [69]. In a phase II single-arm study (*n* = 42), the clinical benefit rate in patients with KIT exon 11 mutations at 16 weeks was 37% [70]. In the POETIG study, another phase II trial that used ponatinib at a reduced dose, the clinical benefit rate was 35%, and the median PFS was 86 days [71].

Dasatinib, a potent inhibitor of BCR-ABL, KIT, and SRC family kinases, was assessed in a phase II study in patients with GIST resistant to imatinib. The median PFS and OS were 2 and 19 months, respectively, and the median PFS in wild-type GISTs was 8.4 months [72]. As per current NCCN guidelines, dasatinib may be used for patients with *PDGFRA* exon 18 mutations insensitive to imatinib, including *PDGFRA* D842V mutations [3].

Cabozantinib, a multitargeted TKI targeting KIT, VEGFR-2, MET, and AXL, was assessed in patients with metastatic GIST after treatment with imatinib and sunitinib in the phase II CaboGIST study. The median PFS at 12 weeks was 60%. DCR was 82%, with PR in 14% and SD in 68% of patients. The median PFS was 5.5 months, and the median OS was 18.2 months [73].

Crenolanib is a TKI with activity against PDGFR and FLT3 with nanomolar activity against *PDGFRA* D842V mutant GIST. Crenolanib was used in a phase II study and is currently being assessed in a randomized, double-blinded, placebo-controlled phase III trial in advanced and metastatic PDGFRA D842V mutant GIST (CrenoGIST) [74,75,76].

Some molecular data indicate that O6-methylguanine-DNA methyltransferase (MGMT) promoter methylation is prevalent in SDH-deficient GISTs, suggesting sensitivity to alkylating agents. Yebra et al. presented data during the 2020 Annual Meeting of Connective Tissue Oncology Society, which demonstrated the therapeutic vulnerability of SDH-deficient GISTs to the DNA alkylating agent, temozolomide, and a 40% rate of objective responses among five patients treated with this drug. The disease control rate was 100% [77,78]. A phase II study (NCT03556384) is ongoing. Further preclinical and clinical research on SDH-deficient GISTs is needed.

For patients with metastatic GISTs with mutations resistant to imatinib, sunitinib, regorafenib, ripretinib, and avapritinib, referral to a clinical trial is recommended [3].

In patients with limited progression, when standard and investigational therapies fail, re-challenge with a TKI that was previously tolerated and effective for symptom palliation may be considered. Patients who progressed on TKI may experience tumor growth acceleration after TKI discontinuation. In a phase III RIGHT study (*n* = 81), after a median follow-up of 5.2 months, the median PFS was 1.8 months in the imatinib group and 0.9 months in the placebo arm (HR 0.46, 95% CI 0.27–0.78; *p* = 0.005) [79].

**Figure 2:**
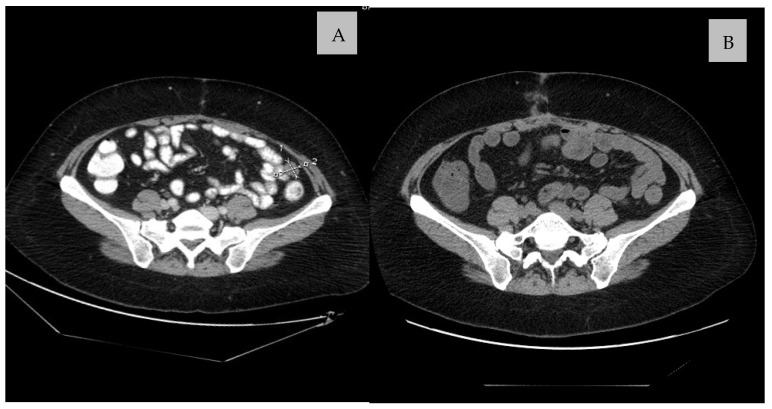
CT scan before (**A**) and after (**B**) resection of residual metastatic lesion in the abdominal cavity in young women with GIST with *KIT* exon 9 deletion mutation arising from the small intestine, after treatment with TKIs.

Surgery may be performed in unresectable and metastatic disease, such as limited disease progression in GIST refractory to imatinib, symptomatic bleeding or obstruction, locally advanced or previously unresectable disease, or low-volume metastatic disease after response to imatinib. Imatinib can be stopped just before surgery and restarted as soon as the patient tolerates oral medications. If the patient is treated with other TKIs than imatinib, such as regorafenib, sunitinib, avapritinib, or ripretinib, the medication should be stopped at least one week prior to surgery and can be restarted based on the clinical assessment [3]. Based on the National Cancer Institute’s Surveillance, Epidemiology, and End Results (SEER) database analysis of patients with GISTs, operative management in young adults with metastases was associated with improved OS (69.5% vs. 53.7%, *p* = 0.04) and GSS (71.5% vs. 56.7%, *p* = 0.03) [29]. The Figure 2 shows CT scans young woman with GIST with KIT exon 9 deletion mutation arising from the small intestine, after treatment with TKIs, who undergo surgical resection of residual lesion in abdominal cavity.

For patients with SDH mutations and SDH deficiency and patients with NF1 mutations, genetic counseling should be advised [3].

## 5. Treatment Tolerability

There are no specific data on the tolerability of drugs used in the treatment of GIST in young adults. Young adults often have overall better organ function than older adults and have fewer comorbidities, take fewer medications, and, therefore, are at lower risk of drug–drug interactions. They may tolerate treatment better than older people. For very young adults, puberty is also a time of significant physical activity and increased physiological changes in body composition, protein binding, and organ function, all of which can affect drug metabolism [38]. Due to the lack of detailed information on the treatment tolerability of individual drugs used in patients with GISTs, the overall data on the toxicity of therapy are summarized below. The most common toxicities of the most frequently used medications are summarized in Table 3.

### 5.1. Imatinib

Drug–drug interactions, compliance, and the genetic variability of metabolizing or drug-resistant enzymes impact the drug concentrations and, as a result, the efficacy and tolerability of imatinib. In adults treated with imatinib, no significant age-related pharmacokinetic differences were observed [80]. The imatinib-related adverse events (TRAE, treatment-related adverse event), hematological and non-hematological, are well known. The most common adverse events (AEs) include fluid retention and edemas (particularly periorbital), abdominal pain, diarrhea, nausea, vomiting, fatigue, rash, and muscle cramps in the fingers and feet. SAEs, such as liver function test abnormalities, lung toxicity, gastrointestinal bleeding, and hematological AEs, have been reported rarely [3].

Side effects may improve with prolonged therapy and can usually be managed with appropriate supportive treatment, but some patients need to discontinue or reduce the dose of the drug. In some studies, imatinib discontinuation has been associated with rapid disease progression. The NCCN GIST guidelines recommend continuing imatinib treatment at a reduced dose if an adverse effect recurs after discontinuation [3]. In the PERSIST-5 study with 5 years of adjuvant imatinib, only 51% completed 5 years of imatinib therapy, and 16% discontinued therapy due to adverse events [35]. If grade 3 neutropenia or thrombocytopenia occurs, the drug should be discontinued until improvement to at least grade 1 (neutrophils > 1.5 × 10^9^/L; platelets > 75 × 10^9^/L) is achieved. Administration of the drug may be resumed at the dose used prior to the adverse event. If the event recurs, the drug should be discontinued and resumed at a lower dose [43]. Cases of acute liver damage (acute hepatitis) have been reported. Liver function should be monitored regularly in patients treated with imatinib. In such cases, prednisolone appears to be useful [80]. The minimum recommended dose of 400 mg of imatinib per day should be used in patients with hepatic impairment. Patients should be aware of this potential complication and know the factors that may increase the risk, such as drug–drug interactions and the effect of certain foods, including alcohol. An increase in the bilirubin concentration to >3 × ULN or an increase in liver transaminases to >5 × ULN requires discontinuation of the drug until bilirubin levels return to <1.5 × ULN and transaminase levels <2.5 × ULN. The patient may continue therapy in reduced doses (from 400 to 300 mg daily, from 600 to 400 mg daily, or from 800 to 600 mg daily) [81]. During treatment with imatinib, other drugs, including protease inhibitors, azole antifungals, selected macrolides, CYP3A4 substrates with a narrow therapeutic window, warfarin, and other coumarin derivatives, should be taken with caution. The patients should avoid the consumption of grapefruit and grapefruit juice and avoid the potent inhibitors of CYP3A. Caffeine may increase the activity of imatinib, and, therefore, caffeine-containing products should not be consumed during treatment with imatinib [81]. Agents that induce CYP3A4, such as carbamazepine, dexamethasone, phenobarbital, phenytoin, rifampicin, and Hypericum perforatum, which may reduce exposure to imatinib and make it less effective, should be avoided [43].

### 5.2. Sunitinib

In 2011, Hutson et al. [82] published the pooled data of 1059 patients who received sunitinib 50 mg/day on an approved regimen of 4 weeks every 2 weeks (*n* = 689) or a continuous dose of 37.5 mg once daily (*n* = 370). A total of 857 (81%) patients were aged <70 years, and 202 (19%) were aged ≥70 years. The median age for the <70 years group was 57 (range 24–69). Treatment tolerability was similar in both groups. Most treatment-related adverse reactions occurred with a similar frequency in both age groups. Adverse effects that occurred significantly less frequently in younger patients were decreased appetite/weight loss, cough, fatigue, edema, and anemia. An AE that occurred more frequently in younger patients was hand-foot syndrome [82]. Hematologic AEs reported during treatment with sunitinib include anemia, thrombocytopenia, and neutropenia. The most commonly reported gastrointestinal AEs were diarrhea, nausea/vomiting, abdominal pain, dyspepsia, and oral ulceration. Treatment of gastrointestinal AEs depends on the severity and includes antiemetic and antidiarrheal drugs [83]. In case of grade 3 diarrhea, sunitinib should be interrupted until improvement to grade 1, and treatment should be resumed at a reduced dose [83,84]. AEs associated with sunitinib treatment often lead to a dose reduction or temporary interruption [84]. The specific AE that may occur during therapy with sunitinib is hypothyroidism, which often does not require treatment discontinuation, and thyroid hormone replacement therapy is sufficient [83,84]. In patients scheduled to undergo major surgery, temporary discontinuation of sunitinib treatment is recommended due to the impaired wound healing observed during sunitinib treatment [83]. Another AE reported by patients is oral mucositis. In the case of grade 3 and 4 mucositis, it is recommended to temporarily stop therapy and reinstitute it with a dose reduction after improvement [84,85]. The next AE often reported by patients treated with sunitinib is hypertension, which should be treated appropriately with antihypertensives. If severe hypertension cannot be controlled with available medications, it may be necessary to discontinue sunitinib treatment temporarily. Treatment may be resumed once hypertension is adequately controlled [83,84]. Cardiac events such as heart failure, myocarditis, decreased left ventricular ejection fraction, cardiomyopathy, and myocardial infarction have also been reported with sunitinib treatment. Treatment with sunitinib should be discontinued if clinical signs or symptoms of heart failure appear [83]. HFS is another AE reported by patients treated with sunitinib, and is most frequently reported as grade 1–2 in 13% and grade 3–4 in 4% of sunitinib-treated patients [86,87]. In order to prevent the occurrence of HFS, patients should be advised to use moisturizing creams from the beginning of sunitinib treatment [84]. Grade 3–4 skin rash is relatively rare but may require temporary interruption or a dose reduction. Treatment, including the use of topical steroids, is recommended. Grade 1–2 skin rash occurs in 14% of sunitinib-treated patients. Skin discoloration has been observed in 30% of sunitinib-treated patients, and alopecia is also an AE reported by sunitinib-treated patients [86,87].

### 5.3. Regorafenib

The safety and efficacy of regorafenib in the treatment of GIST were evaluated in the Phase III GRID trial, where 98% of patients reported AEs of any grade and 72% of patients required dose modification [88]. Treatment termination due to AEs affected 40% of patients treated with regorafenib [53]. These data are similar to a phase II trial of regorafenib in GIST, where 82% of patients required dose modification [52], and to the results of a retrospective analysis of data from 50 GIST patients treated with regorafenib presented by Chamberlain et al. [88]. The most frequent grade 3–4 AEs were HFS and fatigue. The general tolerability profile of regorafenib is quite similar to sorafenib. HFSR (hand-foot skin reactions), hypertension, diarrhea, and fatigue are the most commonly reported AEs observed in clinical trials with both drugs [89]. Treatment of HFSR may include oral or topical analgesics and cool compresses on the skin in grade 1, and topical therapy and oral steroids and/or anesthetics are recommended in grade 2 and 3. Fatigue should be managed by deficiency corrections (vitamin D3, anemia), lifestyle changes (physical exercise, sleep hygiene), nutritional support, or regorafenib dose modification [89].

### 5.4. Avapritinib

In a phase III clinical study with avapritinib in GISTs with exon 18 PDGFRA mutation, the most common AEs reported in at least 10% of patients were edema, nausea, vomiting, decreased appetite, fatigue/asthenia, and cognitive impairment. SAEs reported in this study included anemia (9%), abdominal pain (3%), pleural frostbite (3%), sepsis (3%), gastrointestinal hemorrhage (2%), acute kidney injury (2%), vomiting (2%), pneumonia (1%), and tumor hemorrhage (1%). AEs with fatal outcomes were reported in 3.4% of patients. In total, 49% of patients required dose modifications (reduction or treatment discontinuation). In total, 48% of participants treated with avapritinib experienced grade 1–3 cognitive impairment [90].

### 5.5. Ripretinib

The tolerability of ripretinib was assessed in the phase III INVICTUS study. The most common TEAEs reported in this study in the ripretinib group were alopecia, nausea, diarrhea, fatigue, myalgia, and HFS. The most common treatment-related grade 3 and 4 events were hypertension, fatigue, hypophosphatemia, and increased lipase. Treatment-related SAEs were observed in 9% of patients who received ripretinib and included anemia, heart failure, dyspnea, gastroesophageal reflux disease, and death of unknown cause. TRAEs leading to dose reductions and treatment discontinuation occurred in 6% and 5% of participants. The safety profile of ripretinib was acceptable [54].

### 5.6. Sorafenib

The most common adverse reactions reported in the phase II study conducted by the Korean GIST Study Group were: HFS, skin rash, abdominal pain, and diarrhea; these were grade 1 and 2 adverse reactions, and most of them were reversible. Ten patients required a dose reduction or treatment discontinuation due to intolerance. The most common adverse reactions resulting in dose reductions were: HFS, rash, hypertension, and diarrhea. No toxicity-related deaths were reported. No toxicity-related deaths were observed [60]. Similar grade 3–4 adverse events were also observed in the phase II study published by Kindler et al. [61], including HFS, hypertension, diarrhea, hypophosphatemia, gastrointestinal bleeding, gastrointestinal perforation, thrombosis, and intracranial hemorrhage, and 61% of patients required dose reductions [61].

### 5.7. Pazopanib

In the PAZOGIST study, AEs with grades of at least 3 were observed in 72% of participants in the pazopanib group vs. 17% in the control arm. The most frequently reported AE was hypertension, reported by 38% of the participants. In total, 26% of the patients reported treatment-related SAEs [57].

### 5.8. Dasatinib

The most common AEs of dasatinib observed in a prospective phase II study by Zhou et al. were anemia, proteinuria, fatigue, neutropenia, and diarrhea. In total, 6.9% of the participants discontinued dasatinib due to AEs before the first efficacy assessment, and 17.2% of the patients reported grade 1 gastrointestinal bleeding during treatment [91]. Fluid retention events such as pleural effusion in all grades and grade 3 and 4 occurred in 13% and 6%, respectively [92].

### 5.9. Cabozantinib

The most common TRAEs reported in the CaboGIST study were diarrhea, fatigue, hypertension, stomatitis, weight loss, and HFS. Overall, tolerability was similar to that of other TKIs and was controlled by dose modification and supportive care [73].

### 5.10. Ponatinib

The most common grade 3 and 4 AEs observed in the POETIG study were pain, hypertension, an elevation of lipase or gamma-glutamyl transpeptidase levels, and fever. These AEs occurred in 67% of patients [71]. In the phase II study published by Heinrich et al., rash, constipation, fatigue, muscle pain, and headache occurred in at least 40% of patients [70].

### 5.11. Nilotinib

The safety of nilotinib was evaluated in a phase III study by Reichardt et al. The most common AEs in the nilotinib treatment group were: abdominal pain, nausea, anorexia, fatigue, weakness, and anemia. The most frequent grade 3 and 4 AEs were weakness, increased lipase activity, abdominal pain, vomiting, increased alanine aminotransferase activity, anorexia, anemia, headache, and myalgia. Many adverse reactions of nilotinib, such as skin and subcutaneous tissue disorders, gastrointestinal disorders, musculoskeletal disorders, and general disorders, are mild and can recover without medical intervention. Most of the above-listed grade 3 and 4 adverse events caused by nilotinib should be dealt with by a dose adjustment or treatment interruption [93].

### 5.12. Crenolanib

The safety of crenolanib was evaluated in a phase II study. The most common grade 3 and 4 AEs included elevated liver function parameters and anemia [74].

## 6. Fertility and Pregnancy

An additional important topic related to GIST treatment in young people is the impact on fertility and pregnancy. The potential effects of cancer treatments on pregnancy and a patient’s future fertility constitute a significant concern. They may affect the quality of life for patients treated due to cancer and cancer survivors.

Table 4 shows the effects of anticancer treatment for GISTs on fertility, pregnancy, and lactation.

Data about the effects of anticancer treatment in GISTs on fertility, pregnancy, and lactation is limited. Wael et al. assessed the effect of imatinib on the placenta and implantation in a mouse model. Significant changes that may determine fetal growth were observed. They found changes in the epigenetic markers of essential genes imprinted in the placenta and a reduction in the labyrinthine zone and blood vessels in the placenta. Moreover, an effect on placental growth was observed in case of treatment discontinuation before pregnancy. This research may indicate that imatinib has a long-term effect on pregnancy and implantation. More extended drug withdrawal before pregnancy or additional monitoring for possible placental failure should be considered [94].

Pye et al. retrospectively analyzed 180 women who became pregnant during treatment with imatinib. They published data from 125 pregnancies: 71% of the women were exposed during the first trimester of pregnancy, and 26% of the women were exposed throughout the pregnancy. Of these 125 pregnancies, 28% resulted in termination of the pregnancy and 15% in spontaneous abortion. In total, 9.6% of the newborns had fetal abnormalities: hydrocephalus, craniosynostosis, hypoplastic lungs, renal agenesis, evisceration, and scoliosis. Some fetal abnormalities were more frequent than expected in the normal population [95]. The possible effects of imatinib at the fetal–maternal and placental interface have not been studied. Epigenetic changes may be hidden after the birth of a child who previously appeared normal, which may have long-term health consequences [95,96].

The use of tyrosine kinase inhibitors (TKIs) during pregnancy is still uncommon.

Imatinib treatment affects sperm survival and activity, as described in a report that included semen samples from 48 men treated with imatinib for CML [97].

**Table 4:** The effects of anticancer treatment for GISTs on fertility, pregnancy, and lactation.

Drug	Fertility	Pregnancy	Lactation
imatinib [43]	In non-clinical studies, the fertility of male and female rats was not affected, although effects on reproductive parameters were observed. Studies on patients receiving imatinib and its effect on fertility and gametogenesis have not been performed. Patients concerned about their fertility on imatinib treatment should consult with their physician.	There are limited data on the use of imatinib in pregnant women. There have been post-marketing reports of spontaneous abortions and infant congenital anomalies from women who have taken imatinib. However, studies in animals have shown reproductive toxicity, and the potential risk for the fetus is unknown. Imatinib should not be used during pregnancy unless clearly necessary. If used during pregnancy, the patient must be informed of the potential risk to the fetus.	There is limited information on imatinib distribution in human milk. Studies in two breastfeeding women revealed that both imatinib and its active metabolite could be distributed into human milk. The milk plasma ratio studied in a single patient was determined to be 0.5 for imatinib and 0.9 for the metabolite, suggesting the greater distribution of the metabolite into the milk. Considering the combined concentration of imatinib and the metabolite and the maximum daily milk intake by infants, the total exposure would be expected to be low (~10% of a therapeutic dose). However, since the effects of low-dose exposure of the infant to imatinib are unknown, women should not breastfeed during treatment and for at least 15 days after stopping treatment with imatinib.
sunitinib [83]	Based on nonclinical findings, male and female fertility may be compromised by treatment with sunitinib.	There are no studies on pregnant women using sunitinib, and studies in animals have shown reproductive toxicity, including fetal malformations. Sunitinib should not be used during pregnancy or in women not using effective contraception unless the potential benefit justifies the potential risk to the fetus. If sunitinib is used during pregnancy or if the patient becomes pregnant while on treatment with sunitinib, the patient should be apprised of the potential hazard to the fetus.	Sunitinib and/or its metabolites are excreted in rat milk, and it is not known whether sunitinib or its primary active metabolite is excreted in human milk. Because active substances are commonly excreted in human milk and have the potential for severe adverse reactions in breastfeeding infants, women should not breastfeed while taking sunitinib.
regorafenib [51]	There are no data on the effect of regorafenib on human fertility. Results from animal studies indicate that regorafenib can impair male and female fertility.	There are no data on the use of regorafenib in pregnant women. Regorafenib is suspected of causing fetal harm when administered during pregnancy based on its mechanism of action. Regorafenib should not be used during pregnancy unless clearly necessary and after careful consideration of the benefits for the mother and the risk to the fetus.	It is unknown whether regorafenib or its metabolites are excreted in human milk. In rats, regorafenib or its metabolites are excreted in milk. A risk to the breastfed child cannot be excluded. Regorafenib could harm infant growth and development. Breastfeeding must be discontinued during treatment with regorafenib.
ripretinib [98]	There are no data on the effect of ripretinib on human fertility. Based on findings from animal studies, male and female fertility may be compromised by treatment with ripretinib	There are no data on the use of ripretinib in pregnant women. Based on its mechanism of action, ripretinib is suspected of causing fetal harm when administered during pregnancy, and animal studies have shown reproductive toxicity. Ripretinib should not be used during pregnancy unless the woman’s clinical condition requires treatment with ripretinib.	It is unknown whether ripretinib/metabolites are excreted in human milk, and a risk to the breastfed child cannot be excluded. Breastfeeding should be discontinued during treatment with ripretinib and for at least one week after the final dose.
avapritinib [99]	There are no data on the effect of avapritinib on human fertility, and no relevant effects on fertility were observed in a rat fertility study.	There are no data on the use of avapritinib in pregnant women, and studies in animals have shown reproductive toxicity. Avapritinib is not recommended during pregnancy and in women of childbearing potential not using contraception. If avapritinib is used during pregnancy or if the patient becomes pregnant while taking avapritinib, the patient should be advised of the potential risk to the fetus.	It is unknown whether avapritinib/metabolites are excreted in human milk, and a risk to newborns/infants cannot be excluded. Breastfeeding should be discontinued during treatment with avapritinib and for two weeks following the final dose.

## 7. Conclusions

Gastrointestinal stromal tumors are rare in young adults. They may differ from the disease diagnosed in patients above 40 regarding clinical and molecular characteristics. They are more often diagnosed in the emergency setting and are more often wild type, and more frequently harbor mutations other than *KIT/PDGRFA*. The general rules for surgery and systemic therapy are the same as for patients above 40. However, all decisions should include the social and family roles, expected lifetime, quality of life, and plans for having children. Younger patients have fewer comorbidities, fewer contraindications, and surgical and systemic treatment limitations. The treatment strategy should be defined and implemented by the multidisciplinary team in the sites experienced in sarcomas. Molecular testing should be done whenever possible, as the molecular profile may differ from patients above 40 and may influence the choice of systemic therapy. Surgery optimization with the possible use of preoperative treatment to reduce the extension of surgery needs to be considered. Surgery may also be considered for some metastatic GISTs to improve the OS. Participation in clinical trials, especially after the failure of approved systemic therapies, should always be considered.

## Figures and Tables

**Table 1:** Summary of the main characteristics of GISTs in young people.

Characteristics	
clinical	are rare more often develop in the stomach and small intestine are more often diagnosed in emergency settings the primary resistance to imatinib is more common GISTs with *SDH* mutations tend to metastasize, including lymph nodes, less frequently metastasize to the liver, usually grow slowly, and are often resistant to imatinib may be related to hereditary syndromes, such as Carney’s triad or neurofibromatosis type 1
pathological	may have GIST features of children or adults GIST
molecular	more frequent wild type, more frequent mutations related to resistance to imatinib, including SDH mutations

**Table 2:** Summary of the efficacy of drugs approved for treatment of unresectable and metastatic GISTs.

Authors and Type of Study	Drug	mPFS	mOS	ORR (%)
Blanke et al. phase III randomized trial 2008 (NCT00009906) [56]	imatinib 400 mg vs. imatinib 800 mg	18 vs. 20 months	55 vs. 51 months	43 vs. 41
Demetri et al. phase III randomized trial 2006 (NCT00075218)	sunitinib vs. placebo	22.9 vs. 6.0 weeks	72.7 vs. 64.9 weeks	6.6 vs. 0
Demetri et al. [53] phase III randomized trial 2013 (NCT01271712)	regorafenib vs. placebo	4.8 vs. 0.9 months	HR 0.77; *p* = 0.199	75.9 vs. 34.8
Jean-Yves Blay et al. phase III randomized trial 2020 (NCT03353753) [54]	ripretinib	6.3 vs. 1.0 months	15.1 vs. 6.6 months	9
Jones et al. [45] phase I (NCT025085320)	avapritinib (data for patients with PDGFRA D842V mutation)	NR; PFS at 3 months 100%; 6 months 94%, 12 months 81%	NR; estimated OS at 6 months 100%, 12 months 91%, 24 months 81%	88

mPFS—median progression-free survival; mOS—median overall survival; ORR—objective response rate; NR—not reached; HR—hazard ratio; PDGFRA platelet-derived growth factor receptor A.

**Table 3:** Adverse events reported in clinical trials with drugs approved for the treatment of unresectable and metastatic GISTs.

Authors and Type of Study	Drug	Frequency of Drug Related AEs		Most Frequent Drug-Related AEs		SAEs		AEs Leading to Treatment Discontinuation	Frequency of Dose Modifications
		All Grades	Grade at Least 3	All Grades	Grade at Least 3	Any Grade (Frequency)	Most Frequent		
Blanke et al. [56] phase III randomized trial 2008 (NCT00009906)	imatinib	NA	400 mg 43% 800 mg 63%	NA	400 mg: anemia 9%, GI toxicities 9%, neutropenia 7%, cardiac toxicities 6%, hemorrhage 5% 800 mg: GI toxicities 16%, anemia 14%, cardiac toxicities 14%, neutropenia 10%, hemorrhage 11%	NA	NA	Most common 400 mg: rash, edema, GI bleeding 800 mg: edema, nausea, fatigue	400 mg: at least one dose delay 38%; at least one dose reduction 16% 800 mg: at least one dose delay 59%; at least dose reduction 58%
Demetri et al. [47] phase III randomized trial 2006 (NCT00075218)	sunitinib	83%	NA	anemia 62%, neutropenia 53%, thrombocytopenia 41%, fatigue 34%, diarrhea 29%, skin discoloration 25%, nausea 24%	neutropenia 10%, thrombocytopenia 5%, fatigue 5%, anemia 4%, HFS 4%, diarrhea 3%, asthenia 3%, hypertension 3%,	20%	HFS, diarrhea, hypertension	7.2%	NA
Demetri et al. [53] phase III randomized trial 2013 (NCT01271712)	regorafenib	98%	61%	HFS 56%, hypertension 49%, diarrhea 40%	hypertension 23%, HFS 20%, diarrhea 5%	29%	abdominal pain 4%, fever 2%, dehydration 2%	6%	72%
Jean-Yves Blay et al. [54] phase III randomized trial 2020 (NCT03353753)	ripretinib	NA	NA	alopecia 49%, myalgia 28%, nausea 26%, fatigue 26%, HFS 21%, diarrhea 21%	lipase increased 5%, hypertension 4%, fatigue 2%, hypophosphatemia 2%	9%	All SAEs: anemia, cardiac failure, death of unknown cause, dyspnea, fecaloma, GERD, hyperkalemia, hypophosphatemia, nausea, upper GI hemorrhage	5%	NA
Jones et al. [45] phase I (NCT025085320)	avapritinib 300 mg/d	99%	65%	nausea 69%, anemia 56%, diarrhea 47%, fatigue 41%, decreased appetite 38%, periorbital edema 37%	anemia 22%, neutropenia 9%, decreased neutrophile count (9% diarrhea 6%)	26% in safety population	Anemia, pleural effusion, diarrhea, vertigo	12% in safety population, 14% in D842V population	NA

AE—adverse event, GERD—gastroesophageal reflux disease, GI—gastrointestinal, HFS—hand foot skin reaction, NA—not available, SAE—serious AE.
